# Exploring Dynamic Changes in HIV-1 Molecular Transmission Networks and Key Influencing Factors: Cross-Sectional Study

**DOI:** 10.2196/56593

**Published:** 2024-05-29

**Authors:** Yan He, Ying Tang, Qun Hua, Xin Li, You Ge, Yangyang Liu, Rong Tang, Ye Tian, Wei Li

**Affiliations:** 1 Department of Infection Management Nanjing Drum Tower Hospital Nanjing China; 2 Children's Hospital of Nanjing Medical University Nanjing China; 3 Nanjing Municipal Center for Disease Control and Prevention Nanjing China; 4 School of Public Health Southeast University Nanjing China; 5 Nanjing Qixia District Center for Disease Control and Prevention Nanjing China

**Keywords:** HIV, dynamic, molecular transmission network, influence factors, HIV-1, molecular network, real-time analysis, transmission, Nanjing, infection, gene distance, heterosexual, homosexual, dynamic alteration, dynamic alterations, risk factor

## Abstract

**Background:**

The HIV-1 molecular network is an innovative tool, using gene sequences to understand transmission attributes and complementing social and sexual network studies. While previous research focused on static network characteristics, recent studies’ emphasis on dynamic features enhances our understanding of real-time changes, offering insights for targeted interventions and efficient allocation of public health resources.

**Objective:**

This study aims to identify the dynamic changes occurring in HIV-1 molecular transmission networks and analyze the primary influencing factors driving the dynamics of HIV-1 molecular networks.

**Methods:**

We analyzed and compared the dynamic changes in the molecular network over a specific time period between the baseline and observed end point. The primary factors influencing the dynamic changes in the HIV-1 molecular network were identified through univariate analysis and multivariate analysis.

**Results:**

A total of 955 HIV-1 polymerase fragments were successfully amplified from 1013 specimens; CRF01_AE and CRF07_BC were the predominant subtypes, accounting for 40.8% (n=390) and 33.6% (n=321) of the specimens, respectively. Through the analysis and comparison of the basic and terminal molecular networks, it was discovered that 144 sequences constituted static molecular networks, and 487 sequences contributed to the formation of dynamic molecular networks. The findings of the multivariate analysis indicated that the factors occupation as a student, floating population, Han ethnicity, engagement in occasional or multiple sexual partnerships, participation in anal sex, and being single were independent risk factors for the dynamic changes observed in the HIV-1 molecular network, and the odds ratio (OR; 95% CIs) values were 2.63 (1.54-4.47), 1.83 (1.17-2.84), 2.91 (1.09-7.79), 1.75 (1.06-2.90), 4.12 (2.48-6.87), 5.58 (2.43-12.80), and 2.10 (1.25-3.54), respectively. Heterosexuality and homosexuality seem to exhibit protective effects when compared to bisexuality, with OR values of 0.12 (95% CI 0.05-0.32) and 0.26 (95% CI 0.11-0.64), respectively. Additionally, the National Eight-Item score and sex education experience were also identified as protective factors against dynamic changes in the HIV-1 molecular network, with OR values of 0.12 (95% CI 0.05-0.32) and 0.26 (95% CI 0.11-0.64), respectively.

**Conclusions:**

The HIV-1 molecular network analysis showed 144 sequences in static networks and 487 in dynamic networks. Multivariate analysis revealed that occupation as a student, floating population, Han ethnicity, and risky sexual behavior were independent risk factors for dynamic changes, while heterosexuality and homosexuality were protective compared to bisexuality. A higher National Eight-Item score and sex education experience were also protective factors. The identification of HIV dynamic molecular networks has provided valuable insights into the characteristics of individuals undergoing dynamic alterations. These findings contribute to a better understanding of HIV-1 transmission dynamics and could inform targeted prevention strategies.

## Introduction

AIDS, a sexually transmitted disease, is significantly influenced by the social and sexual network structure and characteristics of people living with HIV [1,2]. Understanding the social and sexual network structure of individuals infected with HIV is important for public health, as it facilitates comprehension of transmission dynamics within the population and informs targeted prevention and control measures [3-5]. Prior research examining the social and sexual network structure of people living with HIV has predominantly used field epidemiological techniques, including questionnaire surveys, peer tracing, and community follow-ups, to delineate the attributes of social and sexual transmission networks [6-9]. However, the extended latency period of HIV and the time gap between infection and diagnosis create a challenge for social communication networks that rely on self-reported data from individuals who are infected [10]. The quality of self-reported data provided by individuals who are infected may be influenced by various factors such as social discrimination, stigma, and privacy concerns. These factors can create barriers to accurate reporting and may result in underreporting or misrepresentation of information. Consequently, conventional epidemiological investigations, including behavioral surveys, exposure history assessments, and contact tracing, encounter difficulties in analyzing the structure and attributes of HIV transmission networks [11]. Alternative approaches and methodologies may need to be considered to overcome these difficulties and obtain a more comprehensive understanding of the HIV transmission network.

The HIV-1 molecular network has garnered extensive acknowledgment as an innovative technique that uses the gene sequences of individuals who are infected with HIV-1 to examine its transmission attributes [12]. It functions as a valuable supplement to the study of HIV-1 social and sexual networks [13-16]. The implementation and progression of the HIV-1 molecular network has introduced novel viewpoints for the prevention and control of HIV/AIDS [17-20]. The molecular network has been used to assess the efficacy of antiretroviral therapy in preventing secondary HIV transmission [21]. This approach is deemed a precise mechanism for HIV/AIDS prevention and control, enabling a more effective use of public health resources [22]. By identifying individuals or groups who are at a higher risk of transmitting HIV-1, targeted interventions can be implemented to prevent the spread of the virus and improve treatment outcomes. Furthermore, extensive investigations can promptly identify individuals with an undiagnosed infection within the transmission network. Moreover, the implementation of timely interventions among individuals who are uninfected in risk networks can effectively impede further dissemination and spread of HIV-1, thereby holding promising prospects for curtailing the spread of HIV and reducing the incidence of new infections. It becomes possible to optimize prevention efforts and achieve better outcomes in controlling the spread of HIV-1 [23].

Prior research on the impact of molecular networks on HIV risk has predominantly concentrated on static network characteristics, assessing the structural attributes of the network at a particular moment. Factors such as network size, density, and individual characteristics (eg, age, sex, occupation, risky sexual behavior, HIV-related knowledge, and attitudes) have been identified as important considerations in understanding HIV transmission patterns and implementing effective prevention and control measures [24,25].

Recent research has underscored the importance of dynamic features within the HIV molecular network [26,27]. Identified actively growing clusters, despite demographic and risk characteristics that may diverge from the overall population, warrant focused intervention prioritization. The dynamics of cluster growth offer valuable guidance for the allocation and prioritization of public health resources, thereby bolstering the utility of networks in assessing intervention efficacy. These dynamic changes intricately intertwine with the personal characteristics and behaviors of individuals who are infected. Real-time analysis of HIV-1 molecular network characteristics provides a framework for resource allocation toward swiftly evolving molecular clusters. This approach facilitates the identification and comprehension of ongoing transformations within molecular networks, thus enabling prompt interventions and response strategies [26,28]. However, prevailing research on molecular networks in China predominantly focuses on static networks at a single timepoint, with limited exploration of dynamic changes [29-32]. Our study specifically targets newly formed and dynamically growing molecular clusters to elucidate the dynamic changes within HIV-1 molecular transmission networks. By analyzing the primary influencing factors driving these dynamics, we aim to unravel the intricate nature of HIV molecular networks. Ultimately, this research endeavor seeks to empower public health initiatives with the capability to more effectively target HIV spread and implement tailored prevention measures through the active monitoring and analysis of molecular network dynamics.

## Methods

### Participants

From September 1, 2015, to June 30, 2019, this research was conducted in five districts of Nanjing: Qinhuai, Xuanwu, Qixia, Jiangning, and Gulou. The study focused on individuals who were newly diagnosed with an HIV infection during this specific time frame. The Nanjing Center for Disease Control and Prevention was responsible for conducting confirmation testing on all individuals infected with HIV included in the study.

### Questionnaire Survey

A structured questionnaire was used for the one-on-one survey, which took place in a separate room. The privacy of the participants was prioritized throughout the questionnaire survey procedure, and strict measures were implemented to uphold the confidentiality of participants’ personal data. The questionnaire covered various topics related to the participants. These included demographic information such as age, gender, occupation as a student or not, marital status, religion, ethnicity, transmission route, sexual orientation, geographical localities (floating population or not), and sex transmission disease history. Additionally, the questionnaire explored participants’ knowledge and behaviors related to HIV, including condom use, casual sexual partners, multiple sexual partners, anal sex, use of enhancers, experiences with sex education, and HIV/AIDS knowledge, using an 8-item questionnaire (National Eight-Item), which was designed by the Chinese Center for Disease Control and Prevention [33,34] (Multimedia Appendix 1).

### Specimen Collection and Storage

Blood samples were collected from survey participants using EDTA anticoagulant tubes and transported to the Nanjing Center for Disease Control and Prevention, and the samples were processed within 12 hours. Plasma, lymphocyte enrichment solution, and red blood cells were separated and divided into separate tubes. The aliquoted cryopreservation tubes were labeled and stored at –80 °C.

### HIV-1 Polymerase Region Fragment Amplification

The amplification of the HIV-1 virus was executed through the use of reverse transcription–polymerase chain reaction and nested polymerase chain reaction methodologies, in accordance with the protocols described earlier [35]. The amplification process was directed toward a segment of the polymerase (Pol) region of the specimen, with a specific focus on the 1-99 amino acids of the protease region and the 1-254 amino acids of the reverse transcriptase region (HXB2: 2253-3312). The length of the amplified sequence was 1060 base pairs. In cases where the amplified sequence was shorter than 1060 base pairs or samples failed to amplify, reamplification and sequencing were performed. HIV drug resistance was demonstrated as with our previous study [35].

### Identification of HIV-1 Subtypes in Nanjing

The gene sequences that were sequenced with success underwent sorting and splicing procedures, while the possibility of contamination was addressed through the use of the web-based plagiarism detection tool, ElimDupes [36]. This study used the web-based HIV basic local alignment search tool (BLAST) for the identification and genotyping of HIV-1 in Nanjing. Furthermore, gene subtype reference sequences, such as CRF01_AE, CRF07_BC, CRF08_BC, CRF5501_B, B, CRF6701_B, and CRF6801_B, were procured from the HIV sequence database website, and a reference sequence data set was constructed in conjunction with the Nanjing HIV-1 gene sequence data. The gene evolution tree was constructed using an approximately maximum likelihood method in FastTree v2.1 (Morgan N Price) for comparative analysis, and the distribution of HIV-1 genotypes in Nanjing was determined based on the findings of the evolution tree.

### The HIV-1 Molecular Network in Nanjing

An approximately maximum likelihood phylogenetic tree was constructed using FastTree v2.1. The tree was built under the general time reversible + gamma distribution + proportion of invariable sites nucleotide substitution model. The Shimodaira-Hasegawa test was performed to assess the support value for each node in the phylogenetic tree. A support value of 90% was used as the threshold for considering a node well supported. The genetic distance between pairs of sequences was calculated using the Tamura-Nei 93 (TN93) method; a genetic distance threshold ≤0.045 was used to identify potential transmission clusters [37,38]. The HIV-1 molecular network in Nanjing was constructed by using a combination of phylogenetic tree and gene distance (90% + 0.045) methods, which was demonstrated in our previous study [39]. The visual editing of HIV molecular transmission networks was accomplished using Cytoscape 3.10.1 (National Resource for Network Biology), a widely used platform for constructing molecular networks [40,41].

### Identification of Dynamic Changes in HIV-1 Molecular Networks

The molecular network constructed from 2015 to 2017 served as the baseline molecular network (initial time point), while the molecular network observed from 2015 to 2019 represented the observed end point molecular network (final time point).

**Figure 1:**
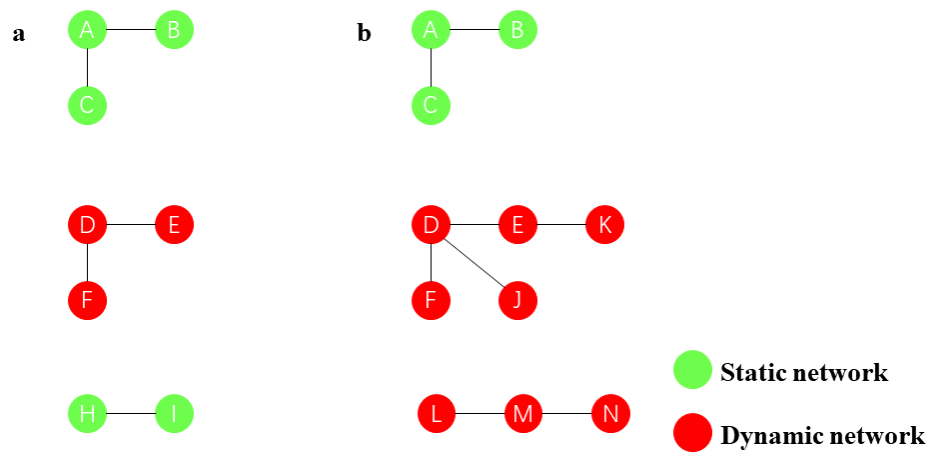
The dynamic changes in molecular networks recognition. (A) shows the baseline molecular network, and (B) shows the observed end point molecular network. The molecular cluster composed of A, B, and C is the unchanged molecular cluster. The molecular cluster composed of H and I do not appear in the observed end point molecular network. The molecular cluster composed of D, E, F, J, and K is a dynamically growing molecular cluster. The molecular cluster composed of L, M, and N is the newly formed molecular cluster. The red spheres represent the dynamic molecular network, while the green spheres represent a statical molecular network.

By comparing the baseline and observed end point molecular networks, the following definitions were derived. The static molecular network consists of unchanged molecular clusters and molecular clusters that do not appear in the observed end point molecular network. This means that the structure and members of these clusters remain constant during the observation period. The dynamic molecular network refers to the newly formed molecular clusters observed in the observed end point molecular network and the dynamically growing molecular clusters based on the baseline molecular network (Figure 1). This indicates that the clusters were formed within the observation period and may continue to grow or change over time.

### Statistical Analysis

EpiData software (v3.1; Odense) was used to construct a database. Two individuals each input the questionnaire data into a computer, and the questionnaires’ completeness were verified using telephone follow-ups or secondary surveys to supplement any missing information. The classification data in this study were expressed as a percentage, and the chi-square test was used to compare various groups. Additionally, multivariate analysis was conducted using logistic regression analysis, with a test level of α=.05.

### Ethics Approval

Written informed consent was obtained from all patients participating in the study, and the research protocol was approved by the Medical Ethics Committee of Zhongda Hospital of Southeast University (2017ZDKYSB045). Our survey is not anonymous; however, all investigators have signed confidentiality agreements to strictly protect the personal privacy information of the participants. The data used for statistical analysis has been deidentified, with the personal privacy information of the investigators removed. Additionally, each participant will receive a compensation of ¥100 (US $15).

## Results

### Subtypes of HIV-1 Pol Fragment Genome in Nanjing From 2015 to 2019

In this study, 955 HIV-1 Pol fragments were successfully amplified from 1013 specimens; the success rate of amplification was 94.27%. Subtype identification revealed that CRF01_AE and CRF07_BC were the predominant subtypes, accounting for 40.8% (n=390) and 33.6% (n=321), respectively. The remaining subtypes included URF, CRF67_01B, CRF68_01B, B, CRF55_01B, CRF08_BC, CRF58_01B, CRF59_01B, CRF87_cpx, and C, which accounted for 9% (n=86), 4.6% (n=44), 3.7% (n=35), 3.5% (n=33), 2.3% (n=22), 1.3% (n=12), 0.6% (n=6), 0.4% (n=4), 0.1% (n=1), and 0.1% (n=1), respectively.

### Identification of HIV-1 Dynamic Molecular Network in Nanjing From 2015 to 2019

**Figure 2:**
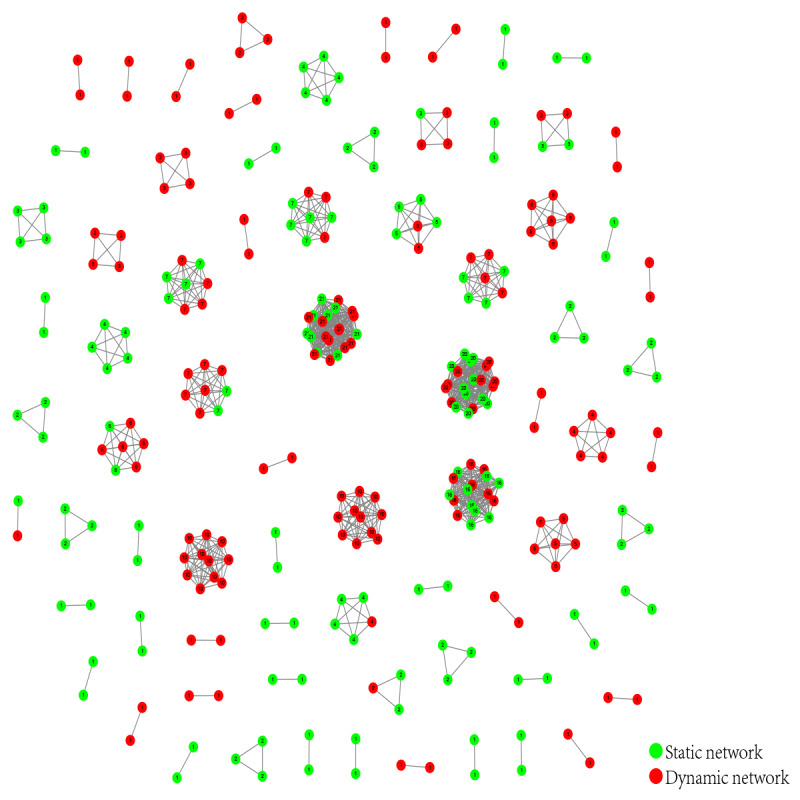
The baseline molecular network. The red spheres represent a dynamic molecular network, while the green spheres represent a statical molecular network. The number in the center of each sphere represents the degree of the molecular network.

The baseline molecular network is the HIV-1 molecular network in Nanjing from 2015 to 2017 (Figure 2). A total of 295 HIV-1 sequences entered the network, with a clustering ratio of 56.8% (295/591), forming 75 molecular clusters. Among them, there were 38 CRF01_AE molecular clusters (122 sequences); 23 CRF07_BC molecular clusters (90 sequences); 2 CRF08_BC molecular clusters (5 sequences); 2 CRF6701_B molecular clusters (23 sequences); and 1 CRF6801_B molecular cluster (17 sequences), 1 CRF5501_B molecular cluster (8 sequences), 3 B subtype molecular clusters (8 sequences), 4 URF molecular clusters (19 sequences), and 1 CRF5901_B molecular cluster (3 sequences).

**Figure 3:**
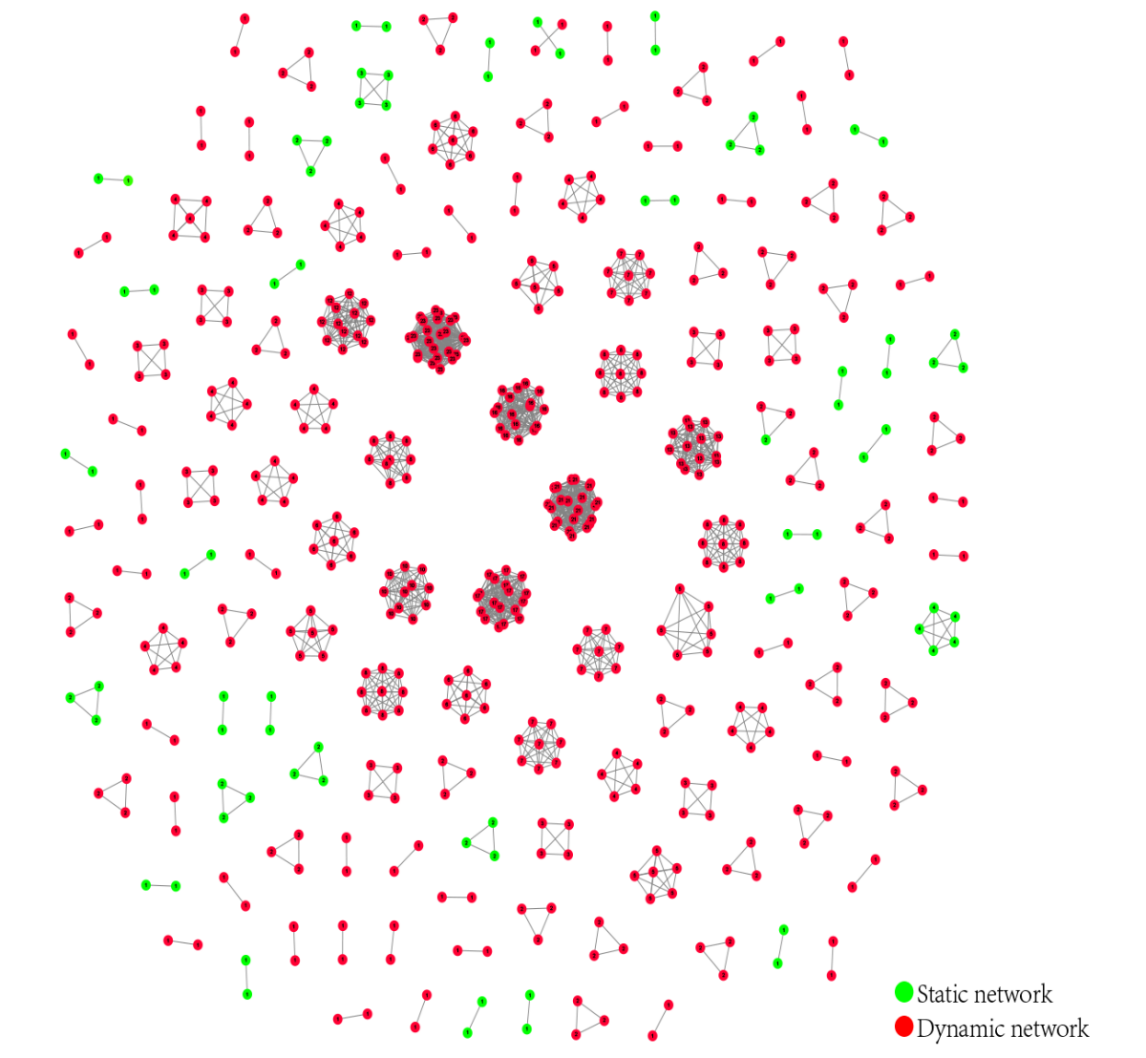
The observed end point molecular network. The red spheres represent a dynamic molecular network, while the green spheres represent a statical molecular network. The number in the center of each sphere represents the degree of the molecular network.

The observation end point molecular network is the HIV-1 molecular network in Nanjing from 2015 to 2019 (Figure 3). A total of 565 HIV-1 sequences were included in the network, with a clustering ratio of 59.2% (565/955), forming 124 molecular clusters. Among them, there were 57 CRF01_AE molecular clusters (233 sequences); 34 CRF07_BC molecular clusters (188 sequences); 2 CRF08_BC molecular clusters (6 sequences); 5 CRF6701_B molecular clusters (34 sequences); and 3 CRF6801_B molecular clusters (19 sequences), 3 CRF5501_B molecular clusters (14 sequences), 8 subtype B molecular clusters (20 sequences), 10 URF molecular clusters (46 sequences), and 2 other molecular clusters including 5 sequences (CRF5801_B 2 pieces, CRF5901_B 3 pieces).

By comparing the baseline and observed end point molecular networks, it was discovered that 144 sequences remained static or disappeared; these sequences formed molecular clusters that constituted a static molecular network. On the other hand, 487 sequences were found in newly formed or dynamically growing molecular clusters, contributing to the formation of dynamic molecular networks within the clusters where these sequences were located. In addition, the remaining 324 gene sequences were excluded from the analysis of the dynamic molecular network because they were absent from both the basic molecular network and the end point molecular network.

### The Impact of General Personal Characteristics on the Composition of Molecular Networks

The molecular network composition (dynamic and static) was taken as the dependent variable for univariate analysis. The findings indicated that the proportion of male individuals who were infected (460/596, 77.2%) entering the dynamic molecular network was marginally higher than that of female individuals (18/26, 69%), although no statistically significant difference was observed. Notably, a significantly higher proportion of infected individuals 20 years and younger entered the dynamic molecular network, with 89.2% (58/65) of such individuals being represented, compared to those older than 20 years (420/557, 75.4%); the difference was statistically significant (*P*=.02). A significantly higher proportion of students who were infected (154/186, 82.8%) were found to have entered the dynamic molecular network compared to nonstudents who were infected (324/436, 74.3%; *P*=.02). The study found significant variations in the proportion of infected individuals with different sexual orientations entering the dynamic molecular network. Specifically, the proportion of bisexual individuals who were infected entering the network was the highest (214/262, 81.7%). Additionally, the proportion of individuals who were infected from the floating population was significantly higher than that of the nonmigrant population (224/277, 80.9% vs 254/345, 73.6%), and the proportion of Han individuals who were infected entering the dynamic molecular network was higher than that of minority individuals who were infected. The study also observed a higher proportion of infection among single individuals, but there were no statistically significant differences in the proportion of people who were infected with different education levels, infection routes, religion, and history of sexuality (Table 1).

**Table 1:** The impact of general personal characteristics on the composition of molecular networks.

Variables		Static molecular network (n=144), n (%)	Dynamic molecular network (n=487), n (%)	Chi-square test (*df*)	*P* value
**Gender**				0.89 (1)	.35
	Female	8 (30.8)	18 (69.2)		
	Male	136 (22.8)	460 (77.2)		
**Age groups (years)**				6.26 (1)	.02
	≤20	7 (10.8)	58 (89.2)		
	>20	137 (24.6)	420 (75.4)		
**Occupation as a student**				5.27 (1)	.02
	No	112 (25.7)	324 (74.3)		
	Yes	32 (17.2)	154 (82.8)		
**Education level**				1.35 (1)	.25
	Below college	52 (26.0)	148 (74.0)		
	College and above	92 (21.8)	330 (78.2)		
**Transmission route**				0.01 (2)	.99
	Homosexual behavior	114 (23.1)	379 (76.9)		
	Heterosexual activity	26 (23.4)	85 (76.6)		
	Other	4 (22.2)	14 (77.8)		
**Sexual orientation**				6.40 (2)	.04
	Heterosexual	25 (24.3)	78 (75.7)		
	Homosexual	71 (27.6)	186 (72.4)		
	Bisexual	48 (18.3)	214 (81.7)		
**Geographical localities**				4.53 (1)	.03
	Permanent residents	91 (26.4)	254 (73.6)		
	Floating population	53 (19.1)	224 (80.9)		
**Ethnicity**				6.38 (1)	.01
	Ethnic minority	10 (45.5)	12 (54.6)		
	Han (Han Chinese)	134 (22.3)	466 (77.7)		
**Religious belief**				0.01 (1)	.95
	No	125 (23.2)	414 (76.8)		
	Yes	19 (22.9)	64 (77.1)		
**Marital status**				7.10 (1)	.008
	Not single	111 (26.2)	312 (73.8)		
	Single	33 (16.6)	166 (83.4)		
**Sex transmission disease history**				0.24 (1)	.63
	No	104 (22.7)	355 (77.3)		
	Yes	40 (24.5)	123 (75.5)		

### The Impact of HIV-1–Related Knowledge and Risky Behaviors on the Molecular Network Composition of People Who Were Infected

There was no significant difference in the proportion of people who were infected entering the dynamic molecular network with different condom use conditions (*P*=.79). However, the proportion of infected persons with casual sexual partners (381/479, 79.5%) was higher than that of patients without casual partners (97/143, 67.8%; *P*=.004). Moreover, the proportion of individuals who reported having multiple sexual partners and engaged in anal sex entering the dynamic molecular network was significantly higher compared to those who did not report such behaviors; these differences were statistically significant. Additionally, the proportion of infected persons with qualified scores in the eight national items who had received sex education entering the dynamic molecular network was significantly lower than the proportion of infected persons with unqualified scores in the National Eight-Item and who had not received sex education. However, there was no statistical difference in the proportion of individuals who were infected who reported using enhancers compared to those who did not (*P*=.96; Table 2).

**Table 2:** The impact of HIV-1 infection knowledge and risky behaviors on molecular network composition.

Variables		Static molecular network (n=144), n (%)	Dynamic molecular network (n=487), n (%)	Chi-square (*df*)	*P* value
**Condom use**				1.05 (3)	.79
	Always	20 (21.7)	72 (78.3)		
	Frequently	54 (22.0)	191 (78.0)		
	Occasionally	53 (25.6)	154 (74.4)		
	Never	17 (21.8)	61 (78.2)		
**Casual sexual partners**				8.49 (1)	.004
	No	46 (32.2)	97 (67.8)		
	Yes	98 (20.5)	381 (79.5)		
**Multiple partners**				19.54 (1)	＜.001
	No	56 (36.1)	99 (63.9)		
	Yes	88 (18.8)	379 (81.2)		
**Anal sex**				8.29 (1)	.004
	No	40 (33.1)	81 (66.9)		
	Yes	104 (20.8)	397 (79.2)		
**Use of enhancers**				0.00 (1)	.96
	No	97 (23.1)	323 (76.9)		
	Yes	47 (23.3)	155 (76.7)		
**Sex education**				4.56 (1)	.03
	No	38 (18.1)	172 (81.9)		
	Yes	106 (25.7)	306 (74.3)		
**National Eight-Item score**				6.39 (1)	.01
	Unqualified	25 (15.8)	133 (84.2)		
	Qualified	119 (25.6)	345 (74.4)		

### Effect of Subtype, Drug Resistance, and Clusters of Differentiation 4 Value on Molecular Network Composition

The statistical analysis revealed a significant difference in the proportion of infected individuals with a clusters of differentiation (CD4) value <500 cells/mm^3^ entering the dynamic molecular network (363/459, 79.1%) compared to those with CD4 value ≥500 cells/mm^3^ (115/163, 70.6%; *P*=.03). However, no statistically significant differences were observed in the proportion of infected individuals with different genotypes or drug resistance entering the dynamic molecular network. Further details can be found in Table 3.

**Table 3:** The effect of subtype, drug resistance, and clusters of differentiation (CD4) value on molecular network composition.

Variables		Static molecular network (n=144), n (%)	Dynamic molecular network (n=487), n (%)	Chi-square (*df*)	*P* value
**Genetic subtypes**				3.00 (2)	.22
	CRF01_AE	62 (25.2)	184 (74.8)		
	CRF07_BC	40 (19.1)	170 (81.0)		
	Other	42 (25.3)	124 (74.7)		
**Drug resistance**				1.79 (1)	.18
	No	120 (22.3)	419 (77.7)		
	Yes	24 (28.9)	59 (71.1)		
**CD4+ (cells/mm3)**				4.92 (1)	.03
	<500	96 (20.9)	363 (79.1)		
	≥500	48 (29.5)	115 (70.6)		

### Logistic Regression Multivariate Analysis

Taking the molecular network status of individuals infected with HIV-1 as the dependent variable (dynamic=1, static=0), the variables with statistical significance in univariate analysis (occupation, age, ethnicity, sexual orientation, floating population, casual sexual partner, multiple sexual partners, National Eight-Item scores, anal intercourse, singleness, sex education experience, and CD4) were included in the multivariate analysis. The assignments were as follows: occupation (student=1, nonstudent=0), age (≤20 years=1, >20 years=0), ethnicity (Han=1, minority=0), sexual orientation (heterosexual=1, same sex=2, bisexual=3), floating population (yes=1, no=0), casual partner (yes=1, no=0), multiple sexual partners (yes=1, no=0), National Eight-Item score (qualified=1, unqualified=0), anal intercourse (yes=1, no=0), marital status (single=1, not single=0), sex education experience (yes=1, no=0), and CD4 (≥500 cells/mm^3^=1, <500 cells/mm3=0).

The findings of the multivariate analysis indicate that various factors, including occupation as a student, migrant status, Han ethnicity, engagement in occasional or multiple sexual partnerships, participation in anal sex, and being single, are independent risk factors for the dynamic changes observed in the HIV-1 molecular network, and the odds ratio (OR) values were 2.63 (95% CI 1.54-4.47), 1.83 (95% CI 1.17-2.84), 2.91 (95% CI 1.09-7.79), 1.75 (95% CI 1.06-2.90), 4.12 (95% CI 2.48-6.87), 5.58 (95% CI 2.43-12.80), and 2.10 (95% CI 1.25-3.54), respectively. Conversely, heterosexuality and homosexuality appear to be protective factors against such changes when compared to bisexuality, with OR values of 0.12 (95% CI 0.05-0.32) and 0.26 (95% CI 0.11-0.64), respectively. Additionally, the National Eight-Item score and experience with sex education were also identified as protective factors against dynamic changes in the HIV-1 molecular network; the OR values were 0.12 (95% CI 0.05-0.32) and 0.26 (95% CI 0.11-0.64), respectively. Further details can be found in Table 4.

**Table 4:** Logistic regression analysis of factors influencing the dynamic changes in HIV-1 molecular networks.

Variables	β (SE)	Wald chi-square (*df*)	*P* values	Odds ratio (95% CI)
Occupation (student)	0.97 (0.27)	12.64 (1)	<.001	2.63 (1.54-4.47)
Heterosexual	–2.10 (0.49)	18.73 (1)	.003	0.12 (0.05-0.32)
Homosexual	–1.35 (0.46)	8.74 (1)	.002	0.26 (0.11-0.64)
Geographical localities (floating population)	0.60 (0.23)	7.14 (1)	.008	1.83 (1.17-2.84)
Ethnicity (Han Chinese)	1.07 (0.50)	4.54 (1)	.03	2.91 (1.09-7.79)
Casual partners (yes)	0.56 (0.26)	4.85 (1)	.03	1.75 (1.06-2.90)
Multiple sexual partners (yes)	1.42 (0.26)	29.62 (1)	＜.001	4.12 (2.48-6.87)
National Eight-Item score (qualified)	–1.58 (0.33)	23.7 (1)	＜.001	0.21 (0.11-0.39)
Anal sex (yes)	1.72 (0.42)	16.50 (1)	<.001	5.58 (2.43-12.80)
Marital status (single)	0.74 (0.27)	7.83 (1)	.005	2.10 (1.25-3.54)
Sex education (yes)	–0.52 (0.24)	4.76 (1)	.03	0.59 (0.37-0.95)
Constant	–0.26 (0.59)	0.19 (1)	.66	0.77

## Discussion

### Principal Findings

The study revealed a diverse distribution of HIV-1 subtypes in Nanjing, with CRF01_AE and CRF07_BC being the predominant subtypes. This observation aligns with the global trend of HIV-1 subtype distribution, with CRF01_AE and CRF07_BC commonly found in Asian regions [39,42]. The identification of various other subtypes, including URFs and less prevalent subtypes, emphasizes the genetic complexity of the HIV epidemic in the studied population.

The dynamic changes in the molecular networks were analyzed to understand the dynamics of HIV-1 transmission [43]. The network’s dynamic changes are indicative of potential transmission associations within it. The addition of new gene sequences can disrupt the existing network and give rise to novel networks, thereby revealing the evolving transmission patterns with greater precision. This approach facilitates the real-time monitoring of the molecular network’s dynamic changes, thereby enabling the prompt identification of rapidly expanding or emerging molecular clusters. Such information can inform targeted interventions and the evaluation of prior prevention and control measures [44].

By conducting a multifactor analysis of the HIV-1 molecular network’s dynamic changes, it was observed that individuals who were bisexual, had multiple sexual partners, had casual sexual partners, and engaged in anal sex were more likely to be present in the dynamic molecular network. Men who have sex with men (MSM) in China may face social and familial pressures that lead them to choose to marry women [45] but engage in extramarital same-sex relationships, thereby acting as a bridge for HIV transmission from high-risk groups to the general population [46]. Bisexual individuals have a higher incidence of unprotected sex, lower risk perception, and weaker awareness of self-protection, making them more susceptible to HIV transmission. Furthermore, individuals constrained by family and involved in fixed marital relationships have limited opportunities to engage with fixed same-sex partners, leading them to seek commercial same-sex services when they have sexual needs. Additionally, individuals who engage in sexual activity with multiple partners are prone to inconsistent condom use in contrast to those who engage in sexual activity with only one partner. The emergence of online social platforms has facilitated casual sexual encounters, resulting in a surge of inadvertent sexual behaviors. The failure to consistently use condoms during casual sexual activity significantly heightens the risk of HIV infection and transmission. The physiological vulnerability of the anus renders anal sex a higher-risk activity, and the lack of condom use during same-sex anal intercourse further exacerbates the risk of infection and transmission [47].

The findings of the analysis indicate that individuals with dynamic network connections are more likely to be affiliated with migrant, single, and student populations. The floating population serves as a significant conduit for HIV transmission across various regions. Furthermore, the population in question predominantly comprises sexually active young adults who spend prolonged periods away from their families, resulting in a dearth of both physical and emotional support. This lack of restraint renders them susceptible to engaging in high-risk behaviors related to HIV. The unattached demographic, unencumbered by familial obligations, exhibits a greater propensity for engaging in precarious conduct, such as engaging in casual sexual liaisons and maintaining multiple sexual partners, thereby heightening their susceptibility to contracting HIV and propagating the virus. In contemporary times, college students have emerged as a crucial cohort for HIV prevention and management. Despite possessing a higher level of education, this group manifests a significant disparity between their awareness and conduct. Their inclination toward seeking novelty coupled with the proliferation of diverse social media and mobile apps, which makes finding sexual partners online extremely convenient, further exacerbates this phenomenon. A study conducted by our research team revealed that a majority of university students infected with HIV contracted the virus through male-to-male sexual behaviors, with mobile apps serving as the primary platform for locating male sexual partners. The male-to-male sexual contacts exhibited a lack of awareness regarding the high prevalence of HIV/AIDS in their population, and they do not consistently use condoms during sexual encounters [48]. Higher National Eight-Item scores and sex education experience also emerged as protective factors, suggesting the potential role of knowledge and awareness in reducing transmission dynamics.

In response to these findings, Nanjing has implemented targeted measures to enhance the accessibility of HIV/AIDS pre-exposure prophylaxis (PrEP) and postexposure prophylaxis (PEP) services among MSM, especially the floating population of Nanjing [49,50]. These measures include improving the availability of PrEP and PEP medication and related health care services, developing educational programs to increase awareness and knowledge about PrEP and PEP, and engaging with MSM communities through community-based organizations and support groups.

### Conclusion

We conducted HIV molecular network analysis in Nanjing from 2015 to 2019, and CRF01_AE and CRF07_BC were identified as the predominant subtypes. Molecular network analysis revealed dynamic changes in the HIV molecular network over time, with 487 sequences contributing to newly formed or dynamically growing molecular clusters. Multivariate analysis confirmed occupation as a student, a floating population, Han ethnicity, engaging in casual or multiple sexual partnerships, participation in anal sex, and being single as independent risk factors for dynamic network changes, while heterosexuality and homosexuality appeared to be protective factors when compared with bisexuality. Additionally, higher National Eight-Item scores and sex education experience were identified as protective factors against dynamic network changes. The findings emphasize the importance of targeted interventions addressing specific risk factors identified in the study. Strategies focused on education, awareness, and behavioral interventions may contribute to stabilizing or reducing the dynamic changes observed in the HIV-1 molecular network. Understanding the intricate dynamics of transmission networks is crucial for designing effective public health measures to control and prevent further spread of HIV.

### Limitations

This study has the following limitations. First, the definition of dynamic network in this study is only based on the comparison of two time point networks, and multiple time point comparisons should be used to discover nascent networks and dynamic growth networks. Second, although we used the HIV Pol gene sequence from 2015 to 2019 to analyze the dynamic changes in the molecular network, the sample size was not large enough. Third, despite conducting the investigation in a one-on-one format, there remains a possibility of recall bias attributable to HIV-related stigma, compounded by the fact that the time of infection is unknown for many individuals.

## Appendix

Multimedia Appendix 1 National Eight-Item questionnaire regarding AIDS prevention.

## Abbreviations

BLAST: basic local alignment search tool
CD: clusters of differentiation
MSM: men who have sex with men
OR: odds ratio
PEP: postexposure prophylaxis
Pol: polymerase
PrEP: pre-exposure prophylaxis
TN93: Tamura-Nei 93

## Data Availability

We have submitted our sequences data to GenBank (submission 2796534), and the data sets are available from WL (weili126@126.com) after the coauthors approve the request.
