# Supplementary material for: Exploring Dynamic Changes in HIV-1 Molecular Transmission Networks and Key Influencing Factors: Cross-Sectional Study
Source: JMIR Public Health Surveill. 2024 May 29;10:e56593. doi: 10.2196/56593 (PMC11170051; doi:10.2196/56593)
Supplement: Multimedia Appendix 1 [file publichealth_v10i1e56593_app1.docx]

“National Eight Articles" regarding AIDS prevention

1. Is AIDS an incurable and severe infectious disease?

A. Yes B. No C. Don't know

2. Is the male homosexual population currently the most seriously affected group by AIDS in China?

A. Yes B. No C. Don't know

3. Can you determine whether a person is infected with AIDS by their appearance?

A. Yes B. No C. Don't know

4. Does having other sexually transmitted diseases increase the risk of contracting AIDS?

A. Yes B. No C. Don't know

5. Can consistently using condoms correctly reduce the risk of contracting and spreading AIDS?

A. Yes B. No C. Don't know

6. Does using new drugs (such as methamphetamine, ecstasy, ketamine, etc.) increase the risk of contracting AIDS?

A. Yes B. No C. Don't know

7. Should one actively seek AIDS testing and counseling after engaging in high-risk behaviors (such as sharing needles for drug use or engaging in unsafe sexual activity)?

A. Yes B. No C. Don't know

8. Is there a legal responsibility for intentionally spreading AIDS?

A. Yes B. No C. Don't know
